# 3D engineered tissue models for studying human-specific infectious viral diseases

**DOI:** 10.1016/j.bioactmat.2022.09.010

**Published:** 2022-09-22

**Authors:** Kyeong Seob Hwang, Eun U Seo, Nakwon Choi, Jongbaeg Kim, Hong Nam Kim

**Affiliations:** a Brain Science Institute, Korea Institute of Science and Technology (KIST), Seoul, 02792, Republic of Korea; b School of Mechanical Engineering, Yonsei University, Seoul, 03722, Republic of Korea; c Yonsei-KIST Convergence Research Institute, Yonsei University, Seoul, 03722, Republic of Korea; d Division of Bio-Medical Science & Technology, KIST School, Korea University of Science and Technology (UST), Seoul, 02792, Republic of Korea; e KU-KIST Graduate School of Converging Science and Technology, Korea University, Seoul, 02841, Republic of Korea

**Keywords:** 3D engineered tissue model, Infectious viral disease, Infection route, Pathology, *In vivo*-mimicking

## Abstract

Viral infections cause damage to various organ systems by inducing organ-specific symptoms or systemic multi-organ damage. Depending on the infection route and virus type, infectious diseases are classified as respiratory, nervous, immune, digestive, or skin infections. Since these infectious diseases can widely spread in the community and their catastrophic effects are severe, identification of their causative agent and mechanisms underlying their pathogenesis is an urgent necessity. Although infection-associated mechanisms have been studied in two-dimensional (2D) cell culture models and animal models, they have shown limitations in organ-specific or human-associated pathogenesis, and the development of a human-organ-mimetic system is required. Recently, three-dimensional (3D) engineered tissue models, which can present human organ-like physiology in terms of the 3D structure, utilization of human-originated cells, recapitulation of physiological stimuli, and tight cell–cell interactions, were developed. Furthermore, recent studies have shown that these models can recapitulate infection-associated pathologies. In this review, we summarized the recent advances in 3D engineered tissue models that mimic organ-specific viral infections. First, we briefly described the limitations of the current 2D and animal models in recapitulating human-specific viral infection pathology. Next, we provided an overview of recently reported viral infection models, focusing particularly on organ-specific infection pathologies. Finally, a future perspective that must be pursued to reconstitute more human-specific infectious diseases is presented.

## Highlights

• 3D *in vitro* models are different from the traditional model in the infection process.
• Human-specific infection research requires a 3D microenvironment and human cells.
• 3D *in vitro* infectious models can be useful for basic research on infectious disease.
• 3D *in vitro* infectious models recapitulate the complex cell-virus-immune interaction.

## Graphical abstract

**Figure:**
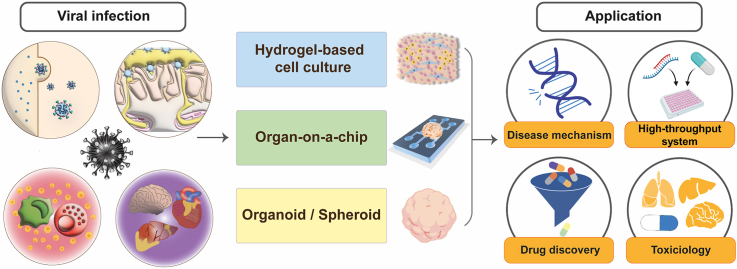

## 1 Introduction

Since the COVID-19 pandemic began, interest in infectious viral diseases has dramatically increased [[1], [2], [3]]. When the virus enters the human body, it uses the cell as a host to multiply its genetic material. The massively replicated virus infects other tissue cells and causes them to malfunction [4]. Furthermore, most viruses are naturally eliminated by the body's innate immune mechanisms, such as recruitment of immune cells to the infection site via the action of interferons. However, if the natural defense system is not sufficiently strong, the viral infection spreads across the human body through blood vessels [[5], [6], [7]]. This causes abnormal cytokine and protein production due to the infection of immune cells, and such immune cell infection causes long-lasting hyper-inflammation, cytokine storm, and immune deficiency. Moreover, the virus transmitted into organs causes cell death, organ dysfunction, and developmental deterioration [[8], [9], [10], [11]].

The sources of viral infection are diverse such as direct or indirect contact with people, animals, insects, and food; the infection routes also vary depending on the organs and virus types [12,13]. For example, viruses targeting the respiratory system makes an entry through human interactions and are transported by inhalation [14]. Previous studies have demonstrated brain infection by using brain organoids [15]. Alimonti et al. prepared a blood–brain barrier (BBB) organoid by aggregating induced pluripotent stem cell (iPSC)-derived brain endothelial cells (i-BEC), neural progenitor cells (i-NP), and mature neurons (i-Ns). The authors confirmed that Zika virus (ZIKV) could cross the BBB and subsequently infect susceptible neural cells [16]. Viral infections exhibit several notable features. Generally, a massive number of individuals are infected, the infection occurs at a localized area, and the time window of breakout and prevalence is seasonal [[17], [18], [19]]. The catastrophic effects of viral diseases are highly dependent on the infection route [20]. Respiratory diseases such as COVID-19, severe acute respiratory syndrome (SARS), and Middle East respiratory syndrome (MERS) can even spread worldwide since they are air-borne diseases [21]. In contrast, infection caused by the human immunodeficiency virus (HIV) is localized in the blood or body fluids and thus does not spread readily [22].

The viral infection route, target organ, pathogenesis, disease progression, and clinical features are diverse; the symptoms are generally not limited to a specific organ, but the infection causes systemic inflammation throughout the body [23]. As seen in the scenario of COVID-19, newly emerging viral diseases can rapidly spread around the world and show high mortality in a short period of time [24]. In such a case, therapeutics must be developed in a timely manner without notable side effects. However, the mechanism underlying the viral infection might be complex, and the current 2D and animal models cannot recapitulate the transmission route, pathogenesis, and subsequent disease progression of such an infection [25]. In addition, the real-time observation of virus-cell interaction is important for identifying therapeutic targets [26]. Therefore, the development of human organ-like engineered systems is essential. The 3D engineered tissue model is a miniaturized human organ model that shows promising capability to model human infectious diseases.

In this review, we summarized the recent advances in 3D engineered tissue models that reflect the features of human-specific infectious diseases. To this end, we first summarized the limitations of the current models in terms of their ability to mimic human physiology and pathology. Next, we provided an overview of the representative 3D engineered tissue models that recapitulate organ-specific infection-associated pathological signatures, including the respiratory, nervous, immune, digestive, and integumentary systems. Finally, the unmet technical needs of the current approaches were discussed. Infectious disease-modeling 3D engineered tissue platforms are believed to help understand human-specific infectious diseases and facilitate the development of therapeutics. In addition, it is possible to predict various viral mutations or the infectivity in 3D human-derived tissue through combined research with an infectious disease and artificial intelligence [27]. Furthermore, this is expected to help obtain more reliable drug test results for numerous drug candidates.

## 2 Conventional models for studying infectious viral diseases: *in vitro* 2D and animal models

**Fig. 1:**
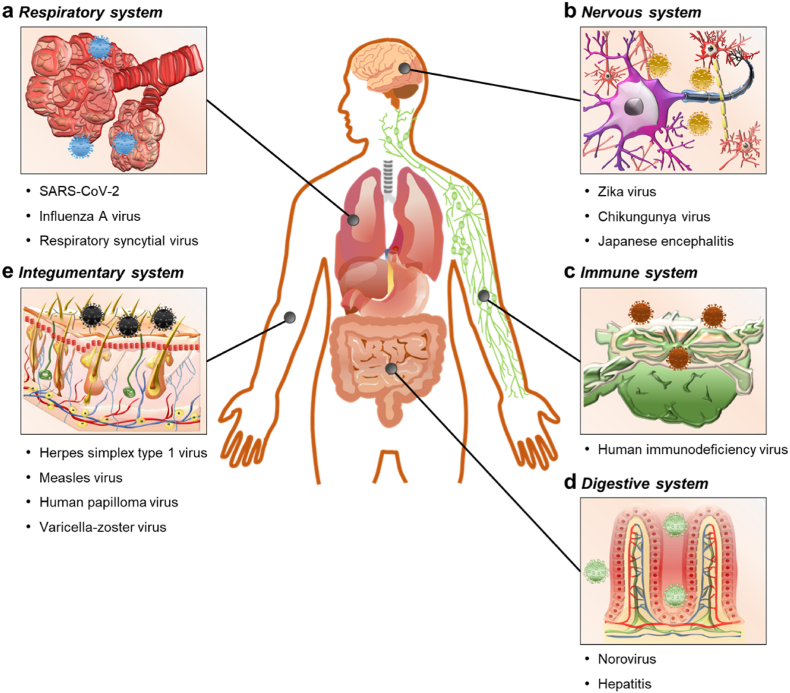
Schematic illustration of viral infection in organ systems. Viral infection in human organs are classified according to infection routes. (a) Respiratory system; SARS-CoV-2, influenza A virus, and respiratory syncytial virus. (b) Nervous system; zika virus, chikungunya virus, and Japanese encephalitis virus. (c) Immune system; human immunodeficiency virus. (d) Digestive system; norovirus, and hepatitis. (e) Integumentary systems; herpes simplex type 1 virus, measles virus, human papilloma virus, and varicella-zoster virus.

**Fig. 2:**
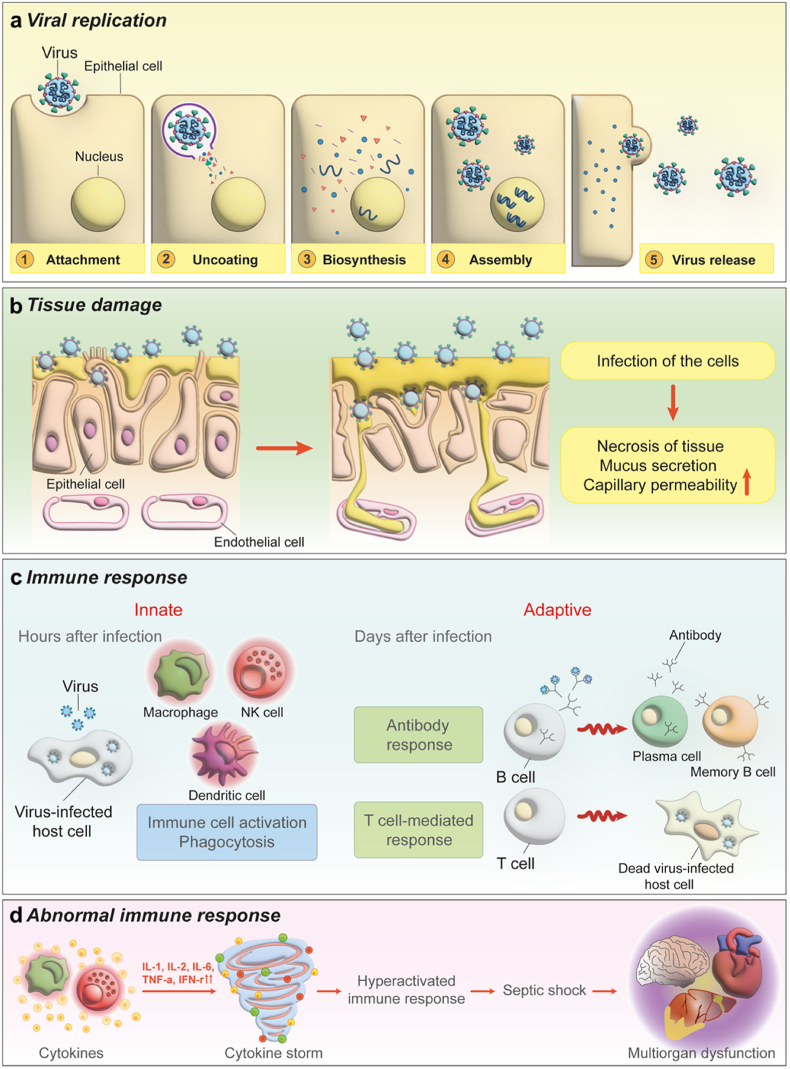
General mechanism underlying viral infection. (a) The general virus replication process. (b) Tissue damage due to viral infection. (c) Types of immune responses after infection. (d) Abnormal immune response process.

Viral infection occurs in the respiratory, nervous, immune, digestive, and integumentary systems (Fig. 1). The Baltimore classification is the most widely used method for classifying viruses (Table 2). However, in 3D *in vitro* models, one important feature is to effectively simulate specific organs and tissues. Thus, it is common practice to categorize and summarize viruses according to the systems in which the virus predominantly infects. Generally, viral entry into the body begins with epithelial cell attachment. Viruses that enter the body gradually damage the tissues, but they activate the immune system. Most infections recover through an activated immune system, but some infections cause an abnormal immune response with severe consequences, such as multi-organ dysfunction (Fig. 2). The mechanism of infection, treatment methods, and symptoms of each organ system vary depending on the virus type. Currently, two models have been widely used: 2D-based cell culture and animal models.

**Table 1:** Classification and characteristics of 3D infectious disease models for each system.

System	Virus	Cell source	Model type	Characteristics	Ref
Respiratory	SARS-CoV-2	HBMVEC, hAT2, Human pluripotent stem cell derived, HPAEpiC, HPMEC, NHBE, Donor	Hydrogel based	The interaction between spike protein and BBB is closely simulated	[166]
				Confirmation of barrier property changes upon the exposure to spike protein	
			Organ on a chip	Confirmation of change in airway cell morphology after the virus infection in 3D	[125]
				Confirmation of virus entry and cytokine changes according to drug treatment concentration	
			Organoid	Simulation of the physiology and pathology of human alveolar cells	[123,124,194]
				Transcriptional changes in infected organoids are closer to real brain tissue than other models	
				Production of organoids from lung cells obtained from donors	
				Identification of cellular composition of the organoid models through single-cell analysis	
				Identification of the inflammatory changes similar to those of an actual infected patient	
				Confirmation of the infection dynamics in various cells in the organoid	
	Influenza A virus	Adult stem cell derived, A549, HSAEpC	Hydrogel based	Fabrication of a mechanical and physical lung tissue-relevant environment by using bioprinting technique	[129,130]
				The difference in immune response according to the infection was confirmed	
				Confirmation of protein expression patterns that are close to those in tissues	
				Mimicry of the *in vivo* immunological phenotypes	
			Organoid	Recapitulation of ciliated cells in organoids that is difficult to implement in a 2D environment	[128]
				Confirmed that the highly contagious viruses also infected the models more than the less contagious viruses	
				Morphologically and functionally close to real tissues	
	RSV	A549, Human pluripotent stem cell-derived	Spheroid	Confirmation of *in vivo*-mimicking phenotype after virus exposure to spheroids (Increased mucin secretion and syncytial formation)	[135]
				A pathologically more relevant model that simulates viral pathogenesis better than the animal model	
			Organoid	This model is not fully matured in contrast to the matured HAE	[137]
				Possible to assess infection at different stages of development	
Nervous	ZIKV	BJ iPSC line, C1-2 line, Human pluripotent stem cell	Organoid	Confirmation of gliogenesis shown *in vivo*	[145,[149], [150], [151], [152], [153]]
				Analysis of organoid degradation and debris formation by viral infection	
				Confirmation of the mechanism underlying infection-induced cell death activation	
				Cost-effective 3D organoid culture system using bioreactor Confirmation of ZIKV infection-induced changes at each stage of neurogenesis	
				Confirmation of DNA methylation changes in astrocyte, neuron, and NPC by ZIKV infection in 3D	
				Confirmation of ZIKV-induced structural changes in organoids A valuable tool for developing anti-ZIKV vaccines	
				Analysis of changes at the cellular level by exposing ZIKV to brain organoids	
				Confirmation of shrinkage of cortical plate and ventricular zone by infection	
				Confirmation of TLR3 pathway activation by ZIKV in hESC-derived cerebral organoids	
				Analyze the relationship between ZIKV and brain microcephaly	
	JEV	Mouse NPC, WA09	Spheroid	Confirmation of activation of interferon signaling pathway by JEV infection in the organoids	[158,159]
				Replication of virus in the organoids with time	
			Organoid	Morphological alteration, impairment in proliferation through JEV infection in neuro-sphere	[159]
				Confirmed that JEV infected astrocyte and oRGCs (outer radial glial cells) during brain development	
	CHIKV	ACS-1013, ACS-1019	Organoid	Confirmation of neurotransmitter excitation pattern in CHIKV-infected organoids depending on whether PD was present The correlation between Parkinson's disease and CHIKV infection was confirmed in 3D cerebral organoids	[164]
Immune	HIV	HMC3, NPC, PBMC, Primary CD4 T cell	Hydrogel based	Intestine-targeted and controlled drug release system	[174,175]
				Simultaneous delivery of three drugs was possible	
				Quantitative analysis, single-cell dynamics analysis of immune cells according to HIV infection in 3D environment	
			Organoid	Interactions between HIV-infected microglia and brain organoids	[173]
				Changes in neuronal viability and cytotoxicity according to HIV infection	
Digestive	Hepatitis V	HepG2-NTCP, PHH, 3T3-J2, KC (Kupffer cell), Huh 7, human hepatocyte	Hydrogel based	Confirmation of tight junction and polarity markers in Huh7 spheroids	[[180], [181], [182], [183], [184]]
				Analysis of specific gene expression according to HCV infection	
				Viral replication and innate immune response were confirmed by infecting HBV and HBD in the SACC-PHH model	
				Analysis of liver-specific transcripts according to co-infection, donor, and time	
				High-throughput system, large-scale genetic screening system	
			Scaffold based	Increased viral replication by HBV in a model constructed using decellularized scaffolds	
				Confirmation of inhibitory effects by drug treatment	
			Organ on a chip	Fabrication of a system to confirm long-term HBV infection in 3D using patient-derived PHH	
				Analysis of immune response according to HBV infection	
				Confirmation of changes in immune factor expression with Kupffer cells	
			Spheroid	Production of spheroids using cellulosic sponge	
				Confirmation of changes similar to *in vivo* and tight junctional localization during HCV infection	
	HuNoV	Caco-2, INT-407 cell	Spheroid	Assessment of various strains of HuNoV viruses using spheroid models	[189,190]
				Implementation of microvilli through 3D cell culture	
				Analysis of RNA replication changes caused by HuNoV exposure	
				Confirmation of total loss of apical microvilli and shortening over time by virus infection	
			Organoid	Elucidation of the interaction between human blood-group antigen (HBGA) type and HuNoV	[191]
Integumentary	HSV	BHK-21, CRFK, HeLa, HMEC-1, HaCaT, PC12, Vero E6	Hydrogel based	Culture of various cells on the 3D bio-printed matrices and subsequent exposure of the Herpes virus	[209,212]
				Confirmed that the bio-printed matrix is suitable for 3D cell culture in terms of cell morphology, polarity, and long-term stability	
				Confirmation of CPE by exposing HSV-1 to 3D cultured PC12 cells	
				Comparison of morphological differences between primary infection and virus reactivation	
	MV	Skin epidermis, dermis	Tissue based	By exposing MV to skin tissues, the measles virus infection appeared more in the dermis	[71]
	HPV	HFK, HFK-31	Hydrogel based	Construction of a model that has undergone epithelial differentiation close to *in vivo* epithelial tissue	[217]

*HBMVEC, Human Brain Microvascular Endothelial Cells; hAT2, Human Lung Alveolar Type 2; HPAEpiC, Human Pulmonary Alveolar Epithelial Cells; HPMEC, Human Pulmonary Microvascular Endothelial Cells; NHBE, Human Bronchial/Tracheal Epithelial Cell; HSAEpC, Human Small Airway Epithelial Cell; PBMC, Peripheral Blood Mononuclear Cell; PHH, Primary Human Hepatocyte.

**Table 2:** Virus classification according to the Baltimore classification.

Class	Characteristics	Examples
Class I	dsDNA virus	Herpes simplex type 1 virus
		Human papilloma virus
Class II	ssDNA virus	–
Class III	dsRNA virus	–
Class IV	(+)ssRNA virus	SARS-CoV-2
		Zika virus
		Japanese encephalitis virus
		Chikungunya virus
		Hepatitis C virus
		Hepatitis E virus
Class V	(−)ssRNA virus	Influenza A virus
		Respiratory syncytial virus
		Hepatitis D virus
		Measles virus
Class VI	retroid/reverse transcribing virus	Human immunodeficiency virus
		Hepatitis B virus

**Fig. 3:**
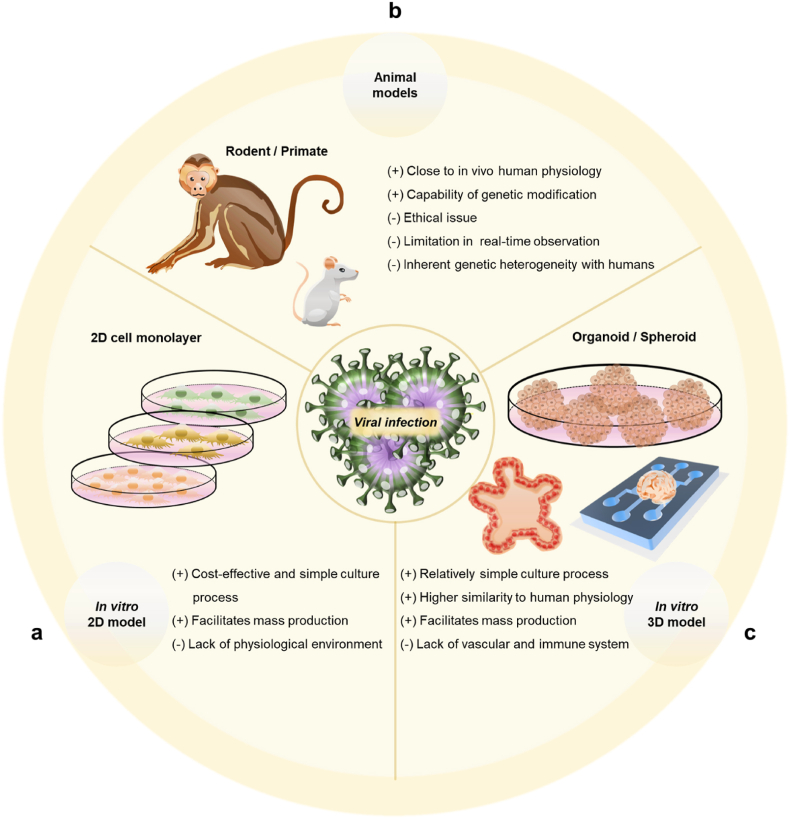
Characteristics of currently used viral infection models. Advantages (+) and disadvantages (−) in the models. (a) *In vitro* 2D models; plastic plate-based cell culture. (b) *In vivo* models; rodents and primates. (c) 3D *in vitro* cell culture models; 3D microfluidics, spheroids, and organoids.

In the case of the 2D *in vitro* models, cells were cultured on a plastic dish and subsequently exposed to the virus (Fig. 3a). This 2D-based cell culture platform is beneficial in terms of ease of control, reproducibility, and cost-efficiency [28,29]. Since cell plating and culture are relatively easy and standardized, various cell types are studied, including stable cell lines, primary human cells, stem cells, and immune cells [[30], [31], [32], [33], [34], [35], [36], [37], [38]]. The platform allows the real-time observation of cellular behaviors, such as viral entry into cells, changes in metabolic activity of cells, activation of immune-related factors, and morphological changes [[39], [40], [41], [42], [43], [44], [45], [46], [47], [48], [49]]. In addition, cells were easily harvested and analyzed for biomolecular changes, including protein and mRNA levels. 2D-based models have been widely used in the identification of viral invasion mechanisms, inhibitors of virus-cell membrane interaction, and potential therapeutics [[50], [51], [52], [53], [54], [55], [56], [57], [58]]. For example, Yang et al. investigated the interaction between the spike glycoprotein and angiotensin-converting enzyme 2 (ACE2) receptor, known as the receptor for SARS-CoV-2, using the A549 cell line [59]. Takahashi et al. studied the effect of dendritic cells on hepatitis C virus (HCV)-infected cells. HCV-infected cells were prepared using Huh 7 cells, and it was confirmed that the amount of type-1 interferon (IFN) increased when co-cultured with dendritic cells, revealing that toll-like receptor 7 signaling was involved in this process [60]. Although these 2D-based cell culture models are useful, they also showed several limitations, including the loss of physiological stimuli, unavailability of diffusion-mediated molecular transport that is essential in 3D tissue, and the inability of tight cell-cell interaction in the 3D environment [61]. In particular, it was more difficult to confirm viral reactivation in the 2D *in vitro* viral infection models compared to the 3D *in vitro* models [35,62]. Therefore, the information obtained may not cover the full spectrum of human infectious diseases.

Research on infectious diseases using experimental animals is mainly conducted in hamsters, mice, rats, ferrets, and monkeys (Fig. 3b) [[63], [64], [65], [66], [67], [68], [69], [70], [71]]. To study contagious diseases that only affect humans, transgenic models carrying human genes are also employed [[72], [73], [74], [75], [76], [77], [78]]. By injecting the virus into an animal model, studies are being actively conducted to check the degree of viral replication, body weight, change in survival rate over time, and levels of cytokine and protein in the animals [[79], [80], [81], [82], [83], [84]]. Hassan et al. produced a SARS-CoV-2 transgenic mouse by delivering human ACE2 to C57BL/6 and BALB/c mouse models, and confirmed that SARS-CoV-2 infection increased viral RNA, lung damage, and weight loss [85]. van den Pol et al. confirmed the mechanism underlying Zika virus infection in a developmental mouse model. The Zika virus spreads within the brain through the axon in this model, and the virus initially infected astrocytes. The model is used to study the mechanisms underlying Zika virus infection at various stages of brain maturation and viral infection of cell types in the brain [85,86]. These animal models can simulate whole-body reactions (e.g., organ-to-organ interaction), systemic inflammation, and infection routes. Although animal models are still the gold standard in infectious disease studies, they also have limitations. For instance, the animal models have genetic heterogeneity with humans, and thus the discrepancy of symptoms are observed [87]. For example, the SARS-CoV-2 murine models failed to show the disease progression to severe symptoms [88]. In addition, the expression of human immune factors in immunodeficient mice is disadvantageous because many human factors cross-react with murine cells, which may lead to unexpected phenotypic changes [89]. The 3D tissue models prepared with the human-originated cells can resolve the genetic heterogeneity issue and thus be exploited in the human-specific virus infection studies [90].

3D engineered tissue models were developed to address these unmet needs. These models included a hydrogel-based 3D cell culture platform, organ-on-a-chip, and organoids (Fig. 3c). Unlike in the *in vivo* system, 2D *in vitro* models exposed to a virus induces cellular infection all at once and an excessive amount of the virus is delivered to the cells [91]. Rosellini et al. showed that the 3D *in vitro* models displayed increased sensitivity to infection by various viruses compared to the 2D *in vitro* models [92]. The 3D engineered tissue models allows for the *in vivo*-like viral exposure via diffusion- and perfusion-mediated transport, demonstrating the *in vivo*-relevance of viral exposure in 3D tissue models [93]. Using these models, the mechanisms underlying infection were analyzed and drugs were screened; these data are being utilized to develop therapeutic agents and vaccines [31,50,94,95].

## 3 3D engineered tissue models for mimicking organ systems

3D engineered tissue models utilize hydrogel-based cell culture [96], 3D spheroids/organoids [97], and organ-on-a-chip [98] (Table 1). These models offer various advantages in analyzing genetic and protein-level changes. Furthermore, the co-existence of multiple types of cells in the confined 3D environment presents an *in vivo*-mimicking tissue structure and phenotype [[99], [100], [101], [102]]. These models facilitate mass production with high reliability; high-throughput and high-content screening are also possible [103,104].

### 3.1 Hydrogel-based 3D tissue model

**Fig. 4:**
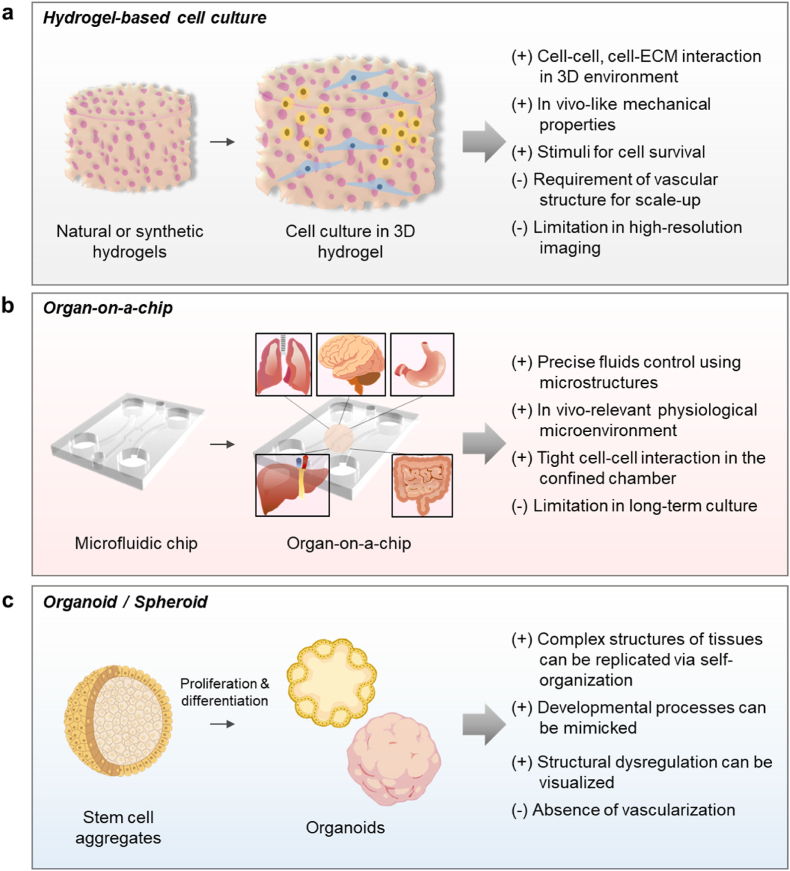
Characteristics of representative 3D *in vitro* models. Advantages (+) and disadvantages (−) of 3D *in vitro* models. (a) The hydrogel-based 3D culture models; cell culture in the 3D hydrogel. (b) The organ-on-chip models; cell culture in the microfluidics. (c) The spheroid/organoid models; culture in aggregated cells (spheroid) and differentiated spheroids (organoid).

The *in vitro* hydrogel-based 3D culture model was produced by culturing various cells within the gelled 3D hydrogel (Fig. 4a) [105]. In general, natural hydrogels such as collagen, Matrigel, hyaluronic acid, alginate, and fibrin, and polymer hydrogels, such as alginate, polyethylene glycol, and GelMa are used. The hydrogel is patterned using 3D printing, chemical treatment, and photopatterning techniques [106]. The hydrogel-based 3D cell culture promotes the complex cell-cell and cell-ECM interactions in the body, provides mechanical support for cells, and forms structural orientation [107]. These characteristics induce notable differences from the cells cultured in a 2D environment, including cellular morphology, abnormal polarization, deviated phenotypic expression, and genotypic features [108].

### 3.2 Organ-on-a-chip

The organ on-a-chip is manufactured by culturing tissue cells within a microfluidic chip (Fig. 4b) [109]. The 3D microfluidic chip are manufactured by soft lithography, 3D printing, and plastic injection molding techniques. In the organ-on-a-chip platform, *in vivo*-mimicking fluidic conditions were simulated, including the interstitial flow, diffusion, and concentration gradient of molecules [110]. By culturing various cells such as the brain, lung, heart, blood vessel, and skin on these microfluidic chips, the physiological and mechanical properties of tissues were simulated. It can implement in-chip blood/airflow [111], breathing motions [112], cardiac contractility and electrophysiology [113]. It stimulates organ functions in the body. This enables efficient and precise drug screening through the dynamic environment within the microfluidic chip, unlike the static environment of the existing plastic-based cell culture [114].

### 3.3 Spheroids/organoids

Spheroids are manufactured using cell aggregation technology using non-adhesive culture wells, hanging drops, spinner flasks, and microfabricated scaffolds (Fig. 4c) [115]. This technology realizes morphological and functional characteristics similar to those of relevant organs in the body by converting single cells into 3D cell aggregates. Spheroids are manufactured using most types of anchorage-dependent cells, and the number and ratio of incorporated cells were controlled [116,117]. In addition, it is possible to manufacture large quantities of spheroids uniformly, which is beneficial for high-throughput screening. However, spheroids do not possess tissue-like features, such as ultrastructure and heterogeneity of cellular distribution.

Organoids are the ‘mini organs’ that are spontaneously formed by the differentiation and self-organization of aggregated stem cells (Fig. 4c). Various types of stem cells are used in the formation of organoids, including embryonic stem cells, adult stem cells, and induced pluripotent stem cells, and their specific lineages are controlled by the growth factors exposed over a time-course [118]. Organoids can mimic complex structures such as laminated layers that are essential in the developmental process, and the disruption or dysregulation of such layered structures were observed in diseased organoid models [90].

## 4 3D *in vitro* infectious viral disease models for pathology

### 4.1 Respiratory system

The most common transmission route of a virus is through the respiratory secretion of an infected person. Such transmissions are largely divided into direct contact among peers or contaminated objects and long-range transmission through droplets and aerosols [119]. The virus invades respiratory organs by binding to receptors on the mucous membrane of the nose and mouth, and the respiratory system is susceptible to viral infection because the mucous membrane is exposed to the external environment. Upper respiratory tract infections (URTIs) often show acute features due to locational characteristics, accounting for most of the respiratory infections [120]. Common URTI symptoms include high fever, headache, sore throat, runny nose, and cough, while symptoms for lower respiaotry tract infections (LRTIs) include cough, sputum, chest pain, wheezing, and hemoptysis. Respiratory viruses are difficult to distinguish medically, and sometimes one virus is classified under various families depending on its genomic structure, infectivity, transmission method, disease severity, and seasonality of circulation [121]. Viral respiratory infections include influenza, coronavirus, and respiratory syncytial virus (RSV). In the following sections, representative cases of 3D models are presented for different virus types.

#### 4.1.1 SARS-CoV-2

SARS-CoV-2 is the causative agent of COVID-19, an ongoing worldwide pandemic, and is known to get internalized into the human respiratory system by binding to ACE2 receptors on the surface of human cells [59]. Since direct manipulation of SARS-CoV-2 is very dangerous and requires specialized virus research facilities, 3D model-based studies are usually conducted using spike proteins.

**Fig. 5:**
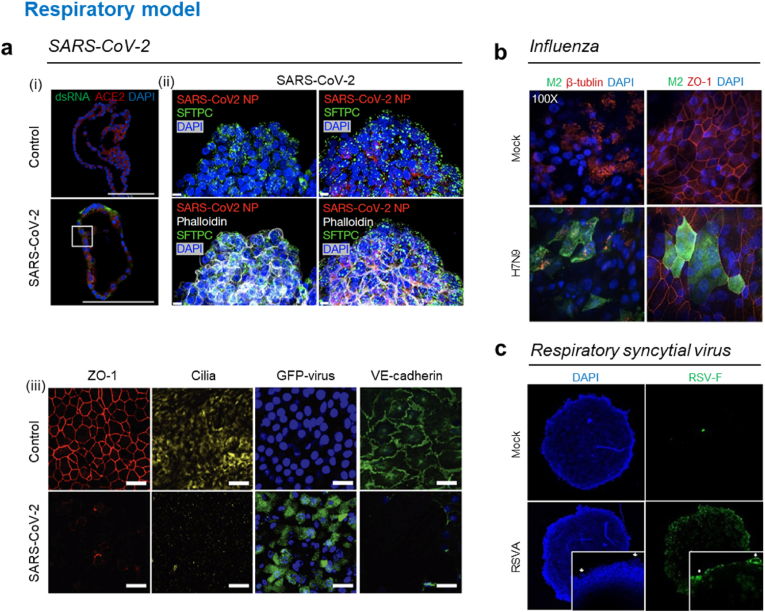
**3D *in vitro* respiratory system model.** (a) SARS-CoV-2-infection models, (i) Representative images of an infected 3D human alveolar model expressing angiotensin-converting enzyme 2. SARS-CoV-2 is identified by viral dsRNA or nucleoprotein. Scale bar, 50 μm. Reproduced from Youk et al. under the terms of the CC-BY license [123]. Copyright 2020, Elsevier. (ii) After SARS-CoV-2 infection in apical-out distal ling organoids (96 hpi), various time-point images through SARS-CoV-2 nucleocapsid protein (NP) and alveolar type 2 (AT2) cell markers in organoids. Scale bar, 10 μm. Reproduced from Salahudeen et al. with permission [124]. Copyright 2020, Springer Nature. (iii) Immunofluorescence staining image of junction changes following exposure to GFP-expressing influenza PR8 (H1N1) virus for 48 h on 3D bronchial-airway-on-a-chip. Scale bar, 50 μm. Reproduced from Si et al. with permission [125]. Copyright 2021, Springer Nature. (b) Influenza virus-infection model, 3D Human Airway Epithelium (HAE) cells infected with H7N9 immunofluorescence staining image according to virus infection. Confocal images, magnification of 100X. Reproduced from Chen et al. Under the terms of CC-BY license [131]. Copyright 2019, Springer Nature. (c) RSV infection-model, The apical surface of the RSV infected tissues (green). White squares highlight groups of infected cells with syncytia formation. Confocal images, magnification of 10X. Reproduced from Saleh et al. with permission [135]. Copyright 2020, Elsevier.

The effect of SARS-CoV-2 infection on the respiratory system has mainly been studied because it first infects the tissues of the respiratory tract. SARS-CoV-2 is known to infect human lung alveolar type 2 (hAT2) cells [122]. Inspired by cell-type specific infection kinetics, Youk et al. developed a 3D hAT2 cell culture model with cyst-like alveolar cell aggregates embedded in Matrigel. When the 3D hAT2 cell aggregates were exposed to the viral solution with SARS-CoV-2 virus for 2 h, the hAT2 cells showed increased expression of interferon-associated and inflammatory genes. The study also showed rapid viral replication, identified using transmission electron microscopy (Fig. 5a (i)) [123]. Salahudden et al. developed a human distal lung organoid and validated its pathogenesis in the distal part of lung tissue. They formed apical-out lung organoids that are relevant to SARS-CoV-2 infection and found that the SARS-CoV-2 virus nanoparticles and dsRNA were predominantly observed in the SCGB1A1^+^ club cells (Fig. 5a (ii)). This result suggested that the target of SARS-CoV-2 infection are club cells [124]. Si et al. utilized an organ-on-a-chip platform to study SARS-CoV-2 infection (Fig. 5a (iii)). They fabricated a double-layered microfluidic device separated by a flexible and transparent porous membrane; human bronchial airway epithelial cells and pulmonary endothelial cells were cultured on either side of the membrane. They found that the clinically relevant dose of the antimalarial drug amodiaquine prohibited infection in the human airway chip model, but other types of antimalarial drugs such as hydroxychloroquine did not show promising effects in inhibiting infection [125].

Buzhdygan et al. studied the effect of the SARS-CoV-2 spike protein on the blood–brain barrier (BBB) using an engineered brain microvasculature model. They found that the human brain endothelial cells in the frontal cortex also express the ACE2 receptor, and the expression level was higher in disease cases such as dementia and hypertension.

#### 4.1.2 Influenza A virus

The influenza virus enters the respiratory system and causes acute febrile respiratory symptoms, including fever and muscle pain [126]. This virus has a short incubation period and is dangerous because it can lead to acute pneumonia in patients with chronic diseases. In particular, influenza virus type A causes explicit disease in humans. Since several types of viruses are available depending on the type of antigens on the virus's surface, researches on the antigen type-specific infection and pathogenesis are required [127]. Zhou et al. developed human airway organoids that contained ciliated cells. They compared the infectivity of influenza viruses depending on the subtypes [128]. For example, the human-infective H7N9/Ah showed higher replication rate compared with poorly human-infective H7N2 virus, and highly human-infective H1N1pdm replicated more robustly than the H1N1sw type. Bhowmick cultured human small airway epithelial cells on a thick chitosan-collagen matrix and studied the effects of the thick hydrogel layer on the function of cultured cells. Cells cultured on the thick hydrogel showed increased expression of aquaporin-5 and cytokeratin-14. When airway epithelial cells cultured on thick hydrogel were exposed to H1N1 and H3N2 influenza viruses, expression of aquaporin-5, cytokeratin-14, and surfactant protein C marker was found elevated only in the H1N1 infection case [129]. Berg et al*.* utilized a 3D bioprinting technique to fabricate an influenza infection model [130]. Although the clustered infection pattern of influenza virus A, which is similar to the natural lung tissue, was observed in the 3D bioprinted model, the 2D monolayer culture model did not show such a pattern. Chen et al. studied human airway epithelial cells (HAEs) in 3D by culturing them and confirmed that exposure to influenza virus (H7N9) causes variation in cilia number and impairment of airways (Fig. 5b) [131]. They found that the replication kinetics of the virus in a 3D microenvironment were different from those observed in conventional 2D-cultured cells. Moreover, antiviral activity was significantly different when an FDA-approved antiviral agent was used to treat the infected 3D HAEs and 2D-cultured cells. These results suggested that the cell culture environment affects infection kinetics and drug responses.

#### 4.1.3 Respiratory syncytial virus

RSV mainly infects infants and children and causes LRTI such as acute pneumonia. The main symptoms are inflammation of the small airways in the lungs and respiratory tract retraction. Previous studies have investigated the relationship between the severity of RSV infection and several genes involved in allergic reactions, innate immunity, and expression of inflammatory cytokines [132]. Currently, no vaccine against RSV has proven to be effective [133]. Although many basic studies on RSV infection and pathogenesis have been conducted using animal models, none of the models produced RSV infection symptoms that were similar to those produced in infants and children [134]. Studies to confirm the effectiveness of vaccines and drug candidates must be conducted using a model with an environment similar to that of humans. Saleh et al. developed spheroids using A549 cells and introduced the RSV virus (Fig. 5c) [135]. RSV-infected A549 spheroids showed mucin accumulation and viral antigen RSV-F dissemination. Geiser et al. confirmed the virus-virus interaction using a commercially available MucilAir™ model in which A549 cells were cultured and investigated the effect of co-infection of RSV with human metapneumovirus. They found that cells infected with RSV were less susceptible to additional infection with human metapneumovirus [136]. Porotto et al. produced pluripotent stem cell-derived lung organoids with branching airways and alveolar structures. They exposed lung organoids to various viruses, such as parainfluenza and RSV, and found that RSV infection caused detachment of infected cells into the lumen of organoids, whereas parainfluenza infection cases did not show such detachment [137].

### 4.2 Nervous system

The infection route in the brain is different from that in the respiratory system because the brain is not in direct contact with the external environment. However, the virus can invade the brain tissue through olfactory nerve transmission or during surgery [138]. Viruses that mainly cause brain infections include the Zika virus, Chikungunya virus and Japanese encephalitis, and brain infections caused by these viruses are largely divided into acute and latent infections [139]. Nervous system infections cause fever, headache, memory loss, seizures, and paralysis. It is difficult to understand the mechanism underlying viral infection in the brain due to the complex structure and connectivity of the brain; thus, the development of a more simple yet robust platform is necessary [15]. 3D *in vitro* brain infection models such as brain organoids and brain-on-a-chip can identify brain tissue damage, such as neuronal development deterioration, neural dysfunction, and cytokine secretion changes [15,[140], [141], [142]]. These brain models are useful in the development of therapeutic solutions for infectious diseases such as microcephaly, Parkinson's disease, chikungunya, and Japanese encephalitis [41,101,143].

#### 4.2.1 Zika virus

**Fig. 6:**
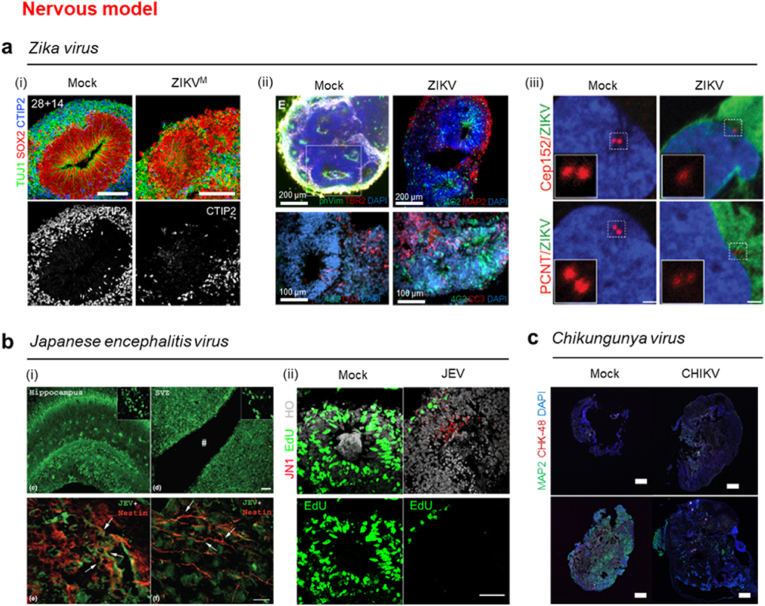
**3D *in vitro* nervous system model.** (a) Zika virus (ZIKV)-infection models, (i) The decrease in thickness of the ventricular zone (VZ) and the neuronal layer of the organoids were observed, resulting in microcephaly-like dysregulation of brain organoid formation. Scale bar, 100 μm. Reproduced from Qian et al. with permission [150]. Copyright 2016, Cell Press. (ii) The cerebral organoids showed significant amount of cell debris and increased expression of cellular apoptosis signals after the exposure to ZIKA virus. Reproduced from Salick et al. with permission [151]. Copyright 2017, MyJove Corp. (iii) ZIKA virus infection in brain organoids causes organoid contraction due to immature differentiation of the neural progenitor cells and changes in the organoid structure. Scale bar, 1 μm. Reproduced from Gabriel et al. with permission [152]. Copyright 2017, Elsevier. (b) JEV infection models, (i) The proliferation ability was decreased, not the death of neural progenitor cells (NPC), in the NPC neurospheres. Scale bar, 100 μm (up), 25 μm (down). Reproduced from Das et al. with permission [158]. Copyright 2008, Wiley-Blackwell. (ii) As the organoids matured, the antiviral immunity effect was more than that of the immature organoids due to increase in the number of glial cells. Scale bar, 50 μm. Reproduced from Zhang et al. under the terms of the CC BY license [159]. Copyright 2018, Nature Portfolio. (c) Chikungunya virus-infection model, more viruses appeared in organoids with Parkinson's disease than in organoids without the disease. Scale bar, 500 μm. Reproduced from Schultz et al. under the terms of the CC BY license [164]. Copyright 2021, MDPI.

The Zika virus infection causes age-dependent symptoms and outcomes [144]. In adults, Zika virus infection leads to Guillain–Barre syndrome, neuropathy, and myelitis [145], while in fetuses, it causes neurodevelopmental disorders such as microcephaly [146]. The brain organoid can simulate the developmental process of the fetal brain. These models show that ZIKV exposure causes reduced growth and viability of brain organoids [141], reduced thickness of the cortical plate [147], a function as entry receptors of the ZIKV-related AXL gene for viral entry mechanism [148], and changes in DNA methylation in brain organoid neurons [149]. Qian et al. produced brain-region-specific organoids such as the forebrain, midbrain, and hypothalamus from human iPSCs (hiPSCs) [150]. These systems provide a quantitative platform to model human diseases and describes microcephalic-like deficits in cortical development. When the Zika virus was exposed to brain organoids, it showed specific tropism toward neural progenitor cells (NPC). A decrease in the thickness of the ventricular zone (VZ) and the neuronal layer of the organoids was observed, resulting in dysregulation of brain organoid formation similar to that observed in microcephaly (Fig. 6a (i)). Salick et al. revealed that cerebral organoids showed significant amounts of cellular debris and increased expression of cellular apoptosis signals after exposure to ZIKV. It was confirmed that neural progenitor cells in cerebral organoids were susceptible to ZIKV infection. It was similar to the phenomena exhibited by microcephaly (Fig. 6a (ii)) [151]. Gabriel et al. showed that ZIKV infection in brain organoids causes damage to neurogenesis by perturbation of centrosomal structure [152]. It resulted in the microcephaly-like organoid contraction due to immature differentiation of neural progenitor cells and changes in organoid structure (Fig. 6a (iii)). Dang et al. confirmed that in the brain organoid model, the innate immune receptor Toll-like receptor 3 (TLR3) was upregulated by ZIKV infection, which caused changes in gene expression related to depletion of the NPC population and the microcephaly phenotypes [153]. In addition, it described a link between ZIKV-mediated TLR3 activation, perturbed cell fate, and a reduction in organoid volume.

#### 4.2.2 Japanese encephalitis virus

The Japanese encephalitis virus (JEV) causes severe neurological infection in the brain [154]. Most diseases caused by JEV infection appear with mild symptoms, but severe symptoms such as mental disorders appear when the virus spreads to the brain [155]. Children under the age of 15 years may die or suffer from complex disabilities, including intellectual, behavioral, or neurological problems such as paralysis, recurrent seizures, or the inability to speak [156]. However, there is still no specific treatment other than prevention using vaccines.

The 3D brain viral infection model facilitates study on antiviral immunity, pathogenesis, and treatment of diseases [157]. Das et al. aggregated mouse NPC to form neurospheres [158]. Exposing JEV into these neurospheres did not kill NPCs, but the proliferation ability of the neurospheres in the NPC decreased. It was confirmed that JEV infection in the dynamic developmental stage of the brain worsened mental function over time (Fig. 6b (i)). Zhang et al. prepared human embryonic stem cell-based telencephalon cortical organoids and exposed them to JEV, which activates interferon signaling [159]. The cells in the organoids showed decreased self-proliferation, increased apoptosis, and exhibited various changes in antiviral immunity depending on the maturation stage of the organoid. Injection of JEV elicits an inflammatory response, increasing the number of glial cells involved in antiviral immunity. In addition, this inflammatory response causes a decrease in Edu + cells, indicating a decrease in proliferation. Therefore, a decrease in Edu + cells is closely related to increased antiviral immunity and glial cells. (Fig. 6b (ii)). These results suggested that 3D brain models can help elucidate the mechanism underlying JEV infection and are expected to be effectively used for the therapeutic development of viral infections.

#### 4.2.3 Chikungunya virus

The chikungunya virus causes inflammation of the brain [160]. CHIKV can be transmitted via mosquitoes and does not spread from person to person [161]. However, there are rare transmission cases which involved handling blood from an infected person [162]. CHIKV infection primarily causes joint pain and can cause neurological diseases such as meningitis, Guillain-Barre syndrome, and nerve paralysis [163]. Accordingly, some 3D models simulate neurological diseases that later occur as complications. Schultz et al. prepared cerebral organoids by obtaining cells from patients with Parkinson's disease [164]. By exposing CHIKV into a 3D *in vitro* brain model, the elicited immune response was observed. This study facilitated analysis of the congenital immune system and confirmed the neurophysiological characteristics through the neurotransmitters generated in the brain model. In addition, it was confirmed that more viruses appeared in organoids with Parkinson's disease than in organoids without the disease (Fig. 6c). Furthermore, upon exposing the chikungunya virus into cerebral organoids, the increase in pro-inflammatory cytokine levels was more in organoids with Parkinson's disease than in organoids without the disease, and the decrease in neurotransmitter levels was also more compared with normal cerebral organoids. These results suggested that viral infection causes morphological and systemic changes such as intrinsic and innate defense dysfunction.

#### 4.2.4 SARS-CoV-2

The coronavirus enters the human body and travels through the blood, and studies have shown that it causes neurological symptoms in some patients [165]. Therefore, model research that simulates the disruptions in the nervous system caused by SARS-CoV-2 infection is actively being conducted. Buzhdygan et al. studied the effect of the SARS-CoV-2 spike protein on the BBB using an engineered brain microvasculature model. The authors found that the human brain endothelial cells in the frontal cortex also expressed the ACE2 receptor, and the expression level was higher in disease cases such as dementia and hypertension. To study the susceptibility of brain tissue to SARS-CoV-2, they formed a microchannel within the hydrogel using microneedles as a template and attached human brain microvascular endothelial cells (hBMEC/D3) to the luminal surface of the microchannel. It was shown that the spike protein of SARS-CoV-2 induced a significant change in the permeability of the brain endothelium, suggesting a potential threat to the BBB [166]. Pellegrini et al. created hiPSC-derived brain organoids and demonstrated that SARS-CoV-2 disrupts blood-cerebrospinal fluid (CSF) barrier function and integrity [167]. These results suggest that the coronavirus not only directly affects neuronal cells, but can also indirectly cause damage by destroying the blood-CSF barrier in the brain. Ramani et al. cultured iPSC-derived human brain organoids for 60 days and then exposed them to coronavirus [168]. The results demonstrated that SARS-CoV-2 targets neurons and that infection causes neuronal cell death.

#### 4.2.5 Human immunodeficiency virus

HIV can affect many organs because it can travel through blood and lymph vessels, destroying immune cells during infection [169]. Based on these characteristics, HIV-infected immune cells are produced mainly in 3D *in vitro* models and then co-cultured with various organ models to conduct experiments [170]. To verify the malfunction of HIV-infected immune cells, a 3D human-derived cell-based organ model is needed as a microenvironment. Gumbs et al. prepared cerebral organoids containing microglia and analyzed the changes after infection with HIV [170]. Existing cerebral organoids had less than 1% microglia, but the authors additionally co-cultured microglia to confirm the interaction between HIV and microglia. The results confirmed that microglia are target cells for HIV and emphasized the need for brain organoids with adequate amounts of microglia in future studies between HIV and the nervous system.

### 4.3 Immune system

Some viruses fundamentally damage the immune system, and the representative infectious disease affecting the immune system is acquired immune deficiency syndrome (AIDS), caused by human immunodeficiency virus (HIV). AIDS suppresses the production of immune cells; thus, the defense mechanism against external pathogens is compromised. HIV infection initially causes high fever, dry cough, lymphadenopathy, skin rash (non-specific rash), myalgia, and a rapid decrease in immune cells, including white blood cells [171]. HIV infection is mainly transmitted through sexual contact, blood transfusion from an infected person, and vertical maternal-fetal transmission. Although research on HIV is underway using several experimental models, drugs that can completely cure it are yet to be developed [172]. The 3D *in vitro* model has shown potential as a drug screening tool to identify the HIV entry process and study the mechanism of treatment.

#### 4.3.1 Human immunodeficiency virus

**Fig. 7:**
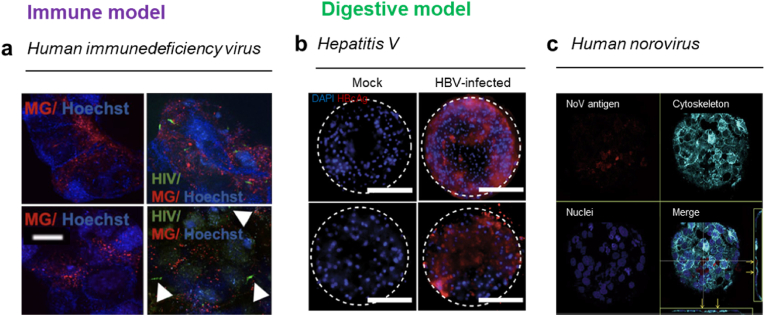
**3D *in vitro* immune and digestive system model.** Immune system, (a) HIV-infection models, characterization of human brain organoids (hBORGs) on day 7 post differentiation. Primary adult brain microglia were infected with HIV-1, membrane labeled, and added to hBORGs. HIV-infected microglia become more attached to hBORGs over time (white arrowheads point to HIV-infected microglia). Scale bar, 200 μm Reproduced from Dos Reis et al. Under the terms of the CC-BY license [173]. Copyright 2020, Springer Nature. Digestive system, (b) Hepatitis virus-infection model, 3D primary human hepatocyte (PHH) cultures for 10 days following infection of 3D cultures with patient-derived HBV. Upper panel shows HBV infection in 3D PHH and lower panel shows HBV infection in 3D spheroid. This shows the suitability of the 3D PHH model. Scale bar, 200 μm. Reproduced from Ortega-Prieto et al. Under the terms of the CC-BY license [182]. Copyright 2018, Springer Nature. (c) HuNoV-infection model, Caco-2 cell aggregates cultured in a rotating wall vessel for 3–4 weeks. Immunofluorescence staining image of HuNoV GII.12/HS206-inoculated INT-407 cells. HuNoV viral capsid protein VP1 was mainly located on surface of cells. Reproduced from Takanashi et al. with permission [189]. Copyright 2013, Springer Nature.

Reis et al. studied the effects of HIV on the nervous system by co-culturing HIV-infected microglia with brain organoids. This model adequately simulates neuroinflammation caused by HIV-1 in terms of the increased production of tumor necrosis factor α (TNF-α) and interleukin 1β (1 L-1β). Furthermore, it is possible to elucidate neuropathological features and molecular dynamics, such as reactive astrocytosis, decreased synaptic density, and neurodegeneration in AIDS (Fig. 7a) [173]. Imle et al. analyzed HIV infection dynamics using an engineered 3D environment and mathematical modeling. Viral replication, infectivity, and cellular motility were confirmed upon exposure to HIV by culturing CD4 T cells in collagen matrix. Furthermore, by adjusting the matrix density by modulating collagen concentration, HIV motility was increased in a less dense environment. The importance of the microenvironment has been emphasized in the study of infectivity and viral activity [174]. Siyawamwaya et al. prepared a novel matrix that was efficiently used for anti-HIV-1 drug delivery [175]. In this matrix, three drugs can be added simultaneously, and controlled release of the drugs is possible. The matrix enables reduction in side effects of drugs, and it shows potential as a drug screening tool. Symeonides et al. confirmed that CD4^+^ T cells cultured in a 3D environment showed dynamic morphological changes compared with suspension culture and directed amoeboid migration [176]. Moreover, they showed that T cell-based syncytia can infect nearby uninfected cells through cell fusion. In addition, cells infected with GFP-tagged HIV migrated and infected other cells over time. These results suggested that a 3D hydrogel-based culture system will facilitate the study of the pathogenesis and transmission of the virus.

### 4.4 Digestive system

The digestive system is also a vulnerable route of infection via food intake, accounting for as much number of infections as through the respiratory system. Representative viruses that affect the digestive system include norovirus and various hepatitis viruses. Many studies on these viruses have been conducted using 2D cell culture and animal models [177,178]. However, in these models, some limitations exist in simulating virus-induced intestinal inflammation and non-specific digestive symptoms. In particular, hepatitis B, one of the most common and severe forms of hepatitis, is transmitted only to primates, including humans. Therefore, although the transgenic model is used extensively, this model, too, differs from the actual human organ system [179].

#### 4.4.1 Hepatitis virus

3D models of the digestive system have been used to study chronic inflammation. Zhang et al. showed the potential for preclinical studies of hepatitis B virus (HBV) through a decellularized liver scaffold-based 3D culture system. In particular, it has been demonstrated that the challenge of the existing primary human hepatocytes were overcome by using a 3D novel scaffold, e.g., liver-specific gene expression, higher infection efficiency, and longer infection period [180]. Winer et al. created a novel 3D co-culture model and showed that primary human hepatocytes maintained their phenotype even after long-term culture. In addition, HBV and hepatitis D virus (HDV) co-infection simulated in a 3D co-culture model confirmed a complex interaction between the two viruses. This model, cultured on a microscale, is suitable for high-throughput drug testing and large-scale genetic screening [181]. Ortega-Prieto et al. produced a 3D model that can be cultured for a long time, and their study confirmed that the expression of innate immune markers and host response factors increases after HBV infection. This revealed that sodium taurocholate co-transporting polypeptide (NTCP), which could not be observed in most 2D systems, plays an essential role in HBV entry and is further involved in the HBV life cycle (Fig. 7b) [182]. Molina-Jimenez et al. fabricated a hepatocyte-like polarized system using a Matrigel-embedded 3D culture system and mimicked polarization characteristics that could not be realized in a traditional 2D model. This model demonstrated that the 3D structure affects the expression of hepatitis C virus (HCV)-related receptors, making it more suitable for HCV infection studies [183]. Ananthanarayanan et al. studied HCV infection by mass-producing spheroids using a sponge-based 3D culture system. This model confirmed that Huh 7.5 cells cultured for up to two weeks maintained liver function and allowed for HCV infection. These results suggest that this model could be useful for basic research on HCV biology and pharmaceutical fields such as drug metabolism and drug-drug interaction [184].

#### 4.4.2 Human norovirus

Unlike common viruses, norovirus has increased activity at low temperatures. Jeong et al. showed the increased activity of norovirus and viral replication at low temperatures in Madin-Darby canine kidney (MDCK) cells with temperature controllability [185]. Furthermore, effective vaccines or antiviral drugs against the virus remain to be discovered. The main symptoms are inflammation of the stomach and intestines, resulting in vomiting, diarrhea, abdominal pain, and dehydration [186]. Moreover, the incubation period of the virus is short, and the infection proceeds with only a small amount of viral particles [187]. Norovirus is generally a zoonotic disease that can infect animals; however, some strains of the virus can infect humans also. For this reason, the use of a model in which a human-like environment is established is necessary to study the human norovirus (HuNoV) [188]. Takanashi et al. cultured human intestinal cells for a long time in the rotation wall vessels (RWV) [189]. With the help of fluidic stimulation, microvilli, which could not be identified in the existing 2D environment, were identified (Fig. 7c). Although this model failed to simulate norovirus infection, the culture of human intestinal cells in 3D through a novel culture method and the simulation of the microvilli structure was of significance. Straub et al. exposed norovirus to a 3D model produced by the RWV culture method and confirmed changes such as the increase in viral replication and total loss of apical microvilli, and shortening of apical microvilli over time [190]. It was shown that the symptoms closely simulated the norovirus infection in a natural environment. To simulate the changes caused by HuNoV infection in a 3D intestinal cell culture model is often challenging. Accordingly, Zhang et al. revealed that norovirus and human blood group antigen (HBGA) interact specifically by exposing the human intestinal organoid, prepared through the stem cell line, to norovirus [191]. Depending on the type of HBGA, even the same virus binds to the cell surface and causes infection. In some cases, viral replication did not occur, even when cells were exposed, and infection symptoms did not appear.

#### 4.4.3 SARS-CoV-2

Since SARS-CoV-2 enters through the respiratory tract, the respiratory infection model dominates. However, the virus can reach all the organs through the bloodstream; thus, the effect of SARS-CoV-2 is not limited to the respiratory system. Therefore, SARS-CoV-2 research on various organs is being actively conducted [192,193]. Han et al*.* demonstrated the usefulness of lung and colonic organoids as a high-throughput drug screening tool [194]. The authors screened the FDA-approved drugs and identified entry inhibitors of SARS-CoV-2, such as imatinib, mycophenolic acid, and quinacrine dihydrochloride. Bein et al. fabricated intestinal-on-a-chip and co-cultured patient organoid-derived intestinal epithelium and human vascular endothelium [195]. In this model, the expression level of the ACE2 receptor, which is used by SARS-CoV-2 for cell entry, was high compared to that of other models, and it was verified that the immune response was activated after infection. In addition, the authors confirmed the decreased viral activity of various drugs such as nafamostat, remdesivir, and toremifene. These studies indicate that the coronavirus that enters the respiratory tract also affects the gastrointestinal (GI) system. They emphasized that the intestinal-on-a-chip is a well-recapitulated tool for the pathophysiology of the organ level. Guo et al. fabricated a human-gut-on-a-chip and demonstrated that coronavirus induces an intestinal response in the chip [196]. The authors verified that villi structures formed in the chip like an actual intestine and a matured intestinal barrier. Moreover, it showed that the intestinal barrier was disrupted, and that there were morphological changes because of coronavirus infection. These are applicable models that can fully reflect the physiological properties of human organs and mimic viral infection and transmission.

### 4.5 Integumentary system

Skin viral infection occurs when herpes simplex type 1 virus (HSV-1), measles, or human papiloma virus (HPV), either present in the air or through a viral host, penetrates the skin and spreads [197]. The route of infection are direct inoculation, regional spread from a specific internal focus, and systemic infection [198]. In direct inoculation, an external virus directly penetrates the nucleus of the skin cell. It injects genetic material into the cell, causing cell death and disruption of the immune response. The virus then proliferates from the infiltrated cells and spreads to the periphery, causing the regional spread of infection. In systemic infection, the virus penetrates the skin and infects the internal system of cells along the blood vessels [199,200]. These skin infections can cause symptoms such as pus, blisters, skin sloughing, skin breakdown, and skin necrosis. In the case of skin-related studies, there is a strong limitation in utilizing animal models globally [201]. Therefore, the development of *in vivo* skin-relevant models is necessary to analyze viral infection mechanisms and produce virus-specific therapeutics [202].

The 3D *in vitro* skin models mimic skin structures using extracellular matrix components and multiple types of skin cells [203,204]. The viral infection were initiated when viruses such as herpes, measles, warts, and shingles come in contact with the skin model, similar to the *in vivo* skin infection [94]. The skin model facilitates identification of the degree of cell death, dysregulated differentiation, and inflammatory response according to the viral infection, and the effect of therapeutics [94,[205], [206], [207]].

#### 4.5.1 Herpes simplex type 1 virus

**Fig. 8:**
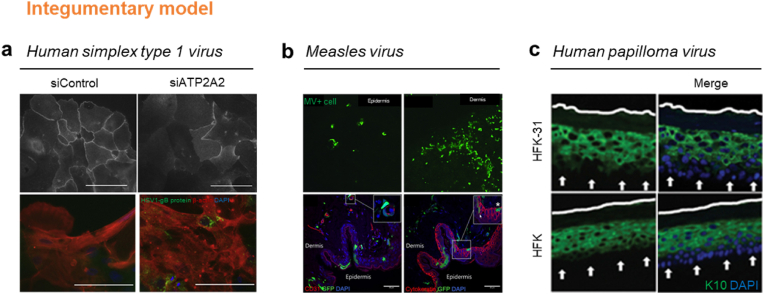
**3D *in vitro* integumentary system model.** (a) Human simple virus (HSV)-infection model, HSV-1 appeared more in the model in which ATP2A2 was absent than that in which ATP2A2 was present. Scale bar, 100 μm. Reproduced from Sato et al. with permission [211]. Copyright 2018, Elsevier. (b) Measles virus-infection model, the measles virus infection appeared more in the dermis sheets as the target site of infection, and the infection rate was high in the dermis even in full skin pieces the skin tissues. Scale bar, 50 μm. Reproduced from Laksono et al. under the terms of the CC BY license [71]. Copyright 2020, PLOS. (c) Human papilloma virus (HPV)-infection model, the expression of the cytokeratin protein involved in the differentiation of keratinocytes by HPV was delayed compared with the general organotypic raft. Reproduced from Anacker et al. with permission [217]. Copyright 2012, MyJove Corp.

HSV-1 causes diseases such as gingivostomatitis, herpes labialis, and herpes keratitis on the skin by direct contact with the skin [208]. The pathogenesis of viral infection is identified by analyzing various disease factors that occur in 3D skin models following viral infection [209]. In addition, it was utilized to confirm changes in the functional properties of the skin barrier and immune response of cells [210]. Sato et al. separated the epidermis and dermis from a piece of human skin and performed organotypic culture by spraying human-based keratinocytes on the dermis tissue (Fig. 8a) [211]. In addition, changes caused by HSV-1 infection were confirmed depending on the presence of ATP2A2, one of the factors of Darier's disease and acrokeratosis verruciformis. They showed that HSV-1 appeared more frequently in a model in which ATP2A2 was absent than when it was present. In addition, in the model with ATP2A2, IFNB1, a protein that suppresses viruses, ISG15, which regulates cytokine secretion, and BAX, which regulates cell death, appear in high levels. The human skin barrier dysfunction model using ATP2A2 is expected to prove the mechanism underlying the occurrence of Kaposi varicelliform eruptions that cause local vesicular eruptions. Hogk et al. fabricated a skin model using human keratinocyte cell line, HaCaT, on human fibroblast-laden collagen [212,213]. To investigate the reactivation of dormant virus, a quiescently infected cell line PC 12 was integrated with the cell-laden collagen matrix. Ultraviolet (UV) light was used to reactivate HSV-1, and activation was confirmed using immunohistochemical detection.

#### 4.5.2 Measles virus

Measles virus (MV) causes severe systemic illness upon contact with the skin [214]. Measles virus infection causes skin rashes all over the skin, making it challenging to analyze region-specific pathogenesis *in vivo*. The infection mechanism and pathogenesis were analyzed using an 3D *in vitro* skin model made with skin biopsy or skin cells [215]. Laksono et al. incubated full skin pieces of epidermal sheets and dermal sheets in 24-well plates [71]. Then, measles virus (MV) was exposed to compare the difference in the infection rate on both the epidermis and dermis (Fig. 8b). Through this, it was confirmed that measles viral infection appeared more significant in the dermis sheets, and the infection rate was high in the dermis, even in full skin pieces from which the epidermis and dermis were enzymatically separated. This suggested that MV infection increased owing to the virus adhesion receptors of Langerhans cells located in the epidermis. In addition, the *in vitro* skin model confirmed that the first viral infection site was the dermis, not the epidermis.

#### 4.5.3 Human papilloma virus

Human papillomavirus (HPV) directly infects the skin, and the disease is transmitted via direct skin-to-skin contact [216]. The HPV infection skin model was fabricated by culturing skin cells as a 3D organotypic raft or in the extracellular matrix and subsequently exposing the HPV virus. Anacker et al. produced a collagen/fibroblast co-culture model, a 3D organotypic raft, by mixing mouse fibroblasts and collagen to confirm the effect of HPV [217]. In the organotypic raft exposed to HPV, the expression of the cytokeratin protein involved in the differentiation of keratinocytes by HPV was delayed compared with that in the general organotypic raft. The proliferation and differentiation of cells increased, resulting in thicker organotypic rafts (Fig. 8c). This model is used to analyze viral pathogenesis and is expected to be the only method for testing antiviral agents.

## 5 Perspectives

Despite advances in 3D *in vitro* infectious viral disease models, there are still issues in simulating viral infections in the body that occur simultaneously with complexity in multiple organ systems. This section presents insights to overcome the limitations of the current 3D *in vitro* infectious viral disease model and its applications.

### 5.1 Necessity of various cell sources for *in vitro* infectious models

The *in vitro* infectious viral disease model was designed to reproduce physiological changes that occur in organs due to viral infection. The prepared infectious viral disease model were used to identify the mechanism underlying the development [218], screen drugs for the treatment [219], and develop vaccines for the prevention, of infectious diseases [194]. However, many viral infection disease models have been developed using animal-based primary cells or cell lines that have different functional and genetic characteristics from humans, limiting the reflection of human-specific pathogenesis [220]. Currently, the hiPSC technique is used. Primarily, the cells derived from hiPSCs are genetically similar to humans and can mimic the functions of internal organs via spontaneous differentiation [221]. In addition, by using iPSC derived from an infectious disease patient, infectious disease-related genes were identified, demonstrating the possibility for precision medicine [222]. In viral infection, the interaction between the tissue and immune system is a crucial consideration [223]. To recapitulate the complex tissue-virus-immune interaction in an *in vitro* infectious disease model, it is essential to incorporate immune cells into the engineered tissue models [224].

### 5.2 Interaction with other organs

Previously, various organ-derived cells, 3D spheroids, and organoids were cultured in a chip and utilized to analyze interactions among cells [225,226]. However, since the virus circulates through blood vessels and reaches various organ systems, it is crucial to check whether viral infection may cause catastrophic effects on multiple organ systems. Therefore, it is necessary to implement a multi-organ system in a chip to accurately simulate a viral infection occurring in the body in an *in vitro* model. Such a system is called human-on-a-chip [227]. In particular, the human-on-a-chip facilitates pharmacokinetic/pharmacodynamics studies [228], intermediate screening for therapeutic efficacy, and assessment of drug toxicity [229]. Consequentially, co-culture of skin and liver tissue, co-culture of liver, heart, and lung tissue, and co-culture of bronchial lung and liver spheroids have been used to confirm the interaction between tissues according to drug treatment [[230], [231], [232]]. For example, Liu et al. confirmed the presence of SARS-CoV-2 through immunohistochemical and immunofluorescence staining of lung, trachea, small intestine, and kidney tissues obtained from COVID-19 patients *in vitro*. SARS-CoV-2 was found in all tissues, confirming that viral infection caused multi-organ injury [233]. This interconnected multi-organ system may facilitate elucidation of the mechanism underlying the development of complex infectious diseases in various organs.

### 5.3 Advanced 3D tissue models

As the biofabrication technology advances, the reproducibility factor is becoming a requirement for the high-throughput screening and commercialization [234]. As an alternative testing model or study platform, the 3D tissue model is robust and reproducible for consistent drug validation and mechanism studies [235]. For this purpose, researchers are aiming to develop a mass-producible platform, such as injection molding-based approaches. Compared to the conventional PDMS-based organ-on-a-chip, the engineered plastic-based chip ensures reproducibility. The scalability is also an important issue for future 3D tissue models. The size limit of the previous fabrication technique was only limited to the millimeter scale due to the inability of oxygen transport in large scale tissue and the maintenance of structural stability. In the case of a 3D *in vitro* model using 3D printing, it is possible to fabricate a model more scalable and consistent than the existing model. This allows for advanced screening of various drug candidates and verifying the effects of drugs [236,237]. Recent 3D bioprinting technology demonstrates the possibility of artificial organs with the real-scale [238] that are potentially applicable to the mechanistic studies of spatio-temporal emergence and the progression of pathogenesis as well as transplantation. There are few studies on a 3D *in vitro* model that simulates the viral infection environment. However, much research has been conducted on robust and scalable 3D *in vitro* models that mimic human tissues and organs [[239], [240], [241]]. In the future, the 3D tissue platforms that offer reproducibility and scalability may revolutionize the study of infectious diseases.

## 6 Summary

In this review article, we summarized the recent advances in the development of 3D viral infection models. These models were fabricated using multiple types of cells in organ microenvironment-mimicking platforms and showed *in vivo*-mimicking pathogenesis and viral replication upon viral exposure. However, there are still many challenges in fully recapitulating human-specific infectious diseases in an engineered system, in terms of human-specific pathogenesis, role of immune cells, organ-to-organ interaction, and AI-based analysis. As observed during the COVID-19 pandemic, humans are always exposed to infectious viral diseases. When an unknown disease emerges, we believe that 3D viral infection models for humans will play a pivotal role in identifying the mechanisms underlying the pathogenesis of infections caused by viruses and in developing appropriate therapeutic methods for such infections.

## Author contribution

^†^K. S. Hwang and E. U. Seo contributed equally to this work. Conceptualization, K.S.H., E.U.S., N.C., J.K., and H.N.K; Investigation, K.S.H., and E.U.S.; Writing – Original Draft, K.S.H., E.U.S.; Writing – Review & Editing, N.C., J.K, and H.N.K.; Funding Acquisition, N.C., J.K., H.N.K.; Supervision, N.C., J.K., and H.N.K.

## Ethics approval and consent to participate

Not applicable.

## Declaration of competing interest

The authors declare they have no competing interests.
